# Use of Pooled State Administrative Data for Mental Health Services Research

**DOI:** 10.1007/s10488-014-0620-y

**Published:** 2015-01-13

**Authors:** Kimberly Eaton Hoagwood, Susan Essock, Joseph Morrissey, Anne Libby, Sheila Donahue, Benjamin Druss, Molly Finnerty, Linda Frisman, Meera Narasimhan, Bradley D. Stein, Jennifer Wisdom, Judy Zerzan

**Affiliations:** 1 Department of Child and Adolescent Psychiatry, New York University Langone School of Medicine, New York, USA; 2 Department of Psychiatry, New York State Psychiatric Institute, Columbia University, New York, USA; 3 Cecil G. Sheps Center for Health Services Research, Department of Health Policy and Management, University of North Carolina at Chapel Hill, Chapel Hill, USA; 4 School of Medicine, University of Colorado Health Sciences Center, Denver, USA; 5 New York State Office of Mental Health (OMH), Albany, USA; 6 Rollins School of Public Health, Emory University, Atlanta, USA; 7 Bureau of Evidence Based Services & Implementation Science, NYS Psychiatric Institute, New York, USA; 8 School of Social Work, University of Connecticut, Storrs, USA; 9 Department of Neuropsychiatry and Behavioral Sciences, School of Medicine, University of South Carolina, Columbia, USA; 10 RAND Corporation, School of Medicine, Pittsburgh, USA; 11 School of Medicine, University of Pittsburgh, Pittsburgh, USA; 12 Office of the Vice President for Research, George Washington University, Washington, USA; 13 Colorado Department of Health Care Policy and Financing, Denver, USA

**Keywords:** States, Mental health, Policy, Systems

## Abstract

State systems are a rich, albeit challenging, laboratory for policy-relevant services research studies. State mental health authorities routinely devote resources to collect data for state planning and reporting purposes. However, these data are rarely used in cross-state comparisons to inform state or federal policy development. In 2008, in response to key recommendations from the National Institute of Mental Health (NIMH) Advisory Council’s “The Road Ahead: Research Partnership to Transform Services,” (http://www.nimh.nih.gov/about/advisory-boards-and-groups/namhc/reports/road-ahead.pdf), NIMH issued a request for applications (RFA) to support studies on the impact of state policy changes on access, cost, quality and outcomes of care for individuals with mental disorders. The purpose of the RFA was to bridge the divide between research and policy by encouraging research that used state administrative data across states, and to address significant state-defined health policy initiatives. Five projects involving eight states were selected through peer review for funding. Projects began in 2009 and were funded for 3 years. This report provides a brief description of the five projects, followed by an analysis of the impact, challenges, and lessons learned from these policy-partnered studies. We conclude by offering suggestions on ways to use state administrative data for informing state health policies, which is especially timely given national and state changes in the structure and financing of healthcare.

State mental health systems routinely collect data for state planning and reporting purposes. All states collect Medicaid data and utilization data for reimbursement that can be used for quality improvement. States are required to report data for special populations served. Many states collect additional data through other systems such as child welfare, corrections, aging, housing, and education programs. In addition, there are administrative data collected through federal agencies [e.g., Centers for Medicare & Medicaid Services (CMS), Social Security Administration (SSA), Substance Abuse and Mental Health Services Administration (SAMHSA), Centers for Disease Control and Prevention (CDC), and Department of Justice (DOJ)] that are available for analyses. Given the vast amount of data and the cumbersomeness of combining data across these entities, such data may be underutilized in the development and monitoring of mental health policies.

This is unfortunate because state systems are a fertile if challenging laboratory for policy-relevant services research studies. Such studies can test the impact of policy decisions on health services delivery. Using these data to not only *inform* but to *create* a set of evidence-based policies has immediate, almost intuitive appeal to policy-makers and healthcare decision-makers (Goldman et al. 2001).

In the development of public policies about healthcare services, however, research evidence can be secondary to interests such as advocacy, political initiatives, media stereotypes, and public opinion (Bowen and Zwi 2005; Dobrow et al. 2004; Waddell et al. 2005). Competing interests are often overlooked in studies examining the use of research evidence by policy-makers (Lomas and Brown 2009). Furthermore, the question of what kind of evidence or information policy-makers actually draw upon when making decisions has been largely unaddressed by researchers (Hyde et al. in press; Soydan and Palinkas 2014).

State mental health systems face an increasingly uncertain environment in which to develop healthcare policies, as the pressures to regulate and manage mental health services are constrained by tighter budgets, changing federal rules, smaller allocation of funds through block grants, and ambiguities about the implementation of the Patient Protection and Affordability Care Act (ACA), passed in 2010 (Hoagwood et al. 2014). State responses to these changes are highlighting the importance of using data systematically to drive healthcare service delivery and decision-making (Gray 2013; Kazdin 2013; Kazdin and Rabbitt 2013; Kelleher 2010). For example, states are struggling with ways to integrate data across their systems and to identify quality indicators for use in monitoring processes and outcomes of care as part of quality improvement initiatives (Institute of Medicine, Committee on Quality of Health Care in America 2006; Institute of Medicine, Committee on Science, Engineering, and Public Policy, National Academy of Sciences, National Academy of Engineering, 2009; Institute of Medicine, Committee on Quality of Health Care in America 2000).

In 2008, in response to key recommendations from the NIMH Advisory Council’s “The Road Ahead: Research Partnership to Transform Services,” (http://www.nimh.nih.gov/about/advisory-boards-and-groups/namhc/reports/road-ahead.pdf), NIMH issued an RFA (RFA-MH-09-050) to support studies on the impact of state policy changes on access, cost, quality and outcomes of care for individuals with mental disorders. The purpose of the RFA was to bridge the gap between research and policy. The RFA required applicants to build their proposed studies around four design considerations that differentiated these studies from typical academic research. First, applicants were required to apply jointly, with one principal investigator representing a state policy perspective, and the other principal investigator a university-based researcher. Second, two states had to be included for comparison purposes. Third, applicants were required to use existing administrative data; no new data collection was authorized so as to reduce costs, model the use of existing administrative data, and, in principle, produce more timely findings. Fourth, each project had to identify study aims that addressed significant mental health policy questions of interest to the host state mental health authority, not just to the researchers, with a research plan that was methodologically rigorous and able to meet peer-review standards.

Five projects were selected for funding through peer review. In this report, the grantees provide a brief description of the five projects, including goals, methods, and their policy or program impacts. In addition, we share the challenges and lessons learned across studies. We conclude by offering suggestions about ways to use state administrative data to inform the kinds of healthcare policy assessments envisioned by the Patient Protection and Affordability Care Act (ACA). Some of the projects’ findings already have undergone peer review, and in those cases, we cite the relevant publications. Other findings are summarized here prior to submission for peer review, hence must be interpreted with appropriate caution.

## Project Overviews

The five projects involved eight states, with comparisons between two states in each project (two projects involved the same two states). Projects began in 2009 and were funded for 3 years; see Table 1 for a summary of studies, methods, major findings, and impacts. The projects differed in terms of populations targeted, data sets used, degree to which the data could be merged, and type of state policy addressed. All of the projects used Medicaid data; in six states, Medicaid data were accessed directly, whereas in SC and WA, databases were used that included Medicaid data. Medicaid data are available in all states and they allow examination of service use across time, providers, and service modalities in ways that would be difficult to determine from other data sources.

**Table 1:** Summary of NIMH R01 grants funded under RFA-MH-09-050 “Use of Pooled State Administrative Data for Policy Relevant Mental Health Services Research”

Pooled states	Principal investigators	Grant title	Research aims	Datasets	Outcome indicators	Key findings
CO-OR	Zerzan and Libby	Sedative hypnotic use by the mentally ill: A Medicaid prescription policy study	Develop a pooled, multi-state Medicaid dataset to study changes in health service utilization associated with policy implementation of sedative hypnotic access restriction policy types (preferred drug lists, prior authorization and cost sharing) in CO and OR	Medicaid	Prescriptions of psychotropics, second generation antipsychotic (SGA) Prescription drug, service utilization, and expenditure Speed and duration of policy impact on sedative hypnotic and SGAprescription fills	Quetiapine was the most frequent SGA in both states (40 % new starts), and for low-dose SGA new starts (55 % CO, 63 % OR). Females had an increased likelihood, and people diagnosed with schizophrenia or anxiety had a decreased likelihood of low-dose quetiapine initiation. Initiation of low-dose quetiapine as a proportion of all SGA initiation and or of quetiapine starts significantly decreased after off-label promotion ended in one state (OR). Cost sharing and Preferred Drug Lists were associated with decreased utilization, but quantity limits were not associated with significant change in prescription rates in both states
CT-WA	Morrissey and Frisman	Community reentry of persons with severe mental illness released from state prison	Assess the impact of expedited Medicaid benefits restoration policies on service utilization and costs among persons with severe mental illness	Medicaid	Mental health service (inpatient and outpatient) use Substance use Arrest and incarceration Cost	Inmates with severe mental illness who received expedited Medicaid benefits were more likely to access mental health services and have shorter time without insurance coverage; no significant effects in criminal justice outcomes and costs
GA-SC	Narasimhan and Druss	Clinical and policy implications of a statewide emergency telepsychiatry program	Evaluate the impact of a statewide telepsychiatry intervention in emergency departments on service utilization and costs	Medicaid All-payor, health data warehouse	Inpatient admission Outpatient follow-up Total cost	Intervention state had low rates of inpatient admission, lower costs, and higher rates of outpatient follow-up than a matched control state
NY-PA	Essock, Donahue and Stein	Evaluating the impact of clinical alerts generated from Medicaid claims data	Using Medicaid claims data to generate clinical flags predicting short-term risk of continued psychiatric hospitalizations	Medicaid	Psychiatric hospitalization Outpatient service use Psychotropic prescription Cost of service	Multiple recent hospitalizations significantly predicted high short-term risk of continued frequent hospitalizations, but absence of recent medication fills and absence of recent outpatient services did not
NY-PA	Wisdom, Hoagwood, Finnerty and Stein	Quality improvement implementation in child mental health: A 2-state comparison	Evaluate the impact of a statewide continuous quality improvement initiative for psychotropic polypharmacy and the effects of prior authorization policies on antipsychotics psychotropic prescription among children and adolescents	Medicaid Area Resource File	Psychotropic polypharmacy Antipsychotics prescription	Polypharmacy patterns were associated with bipolar disorder, older age, specialty mental health services; polypharmacy decreased following clinic participation in the continuous quality improvement initiative; prior authorization policies had a modest but statistically significant effect on decreased antipsychotic use in children aged 6–12

### CO-OR

Libby and Zerzan used Colorado and Oregon Medicaid claims data to measure the extent to which Medicaid prescription drug policies reduced the use of newer sedative hypnotics and increased the unintended use of low-dose second generation atypical antipsychotics (SGA) as possible sedative substitutes. This was an important issue because low-dose antipsychotics may be used off-label (i.e., for other than a Food and Drug Agency (FDA) indication) despite more frequent side effects, compared to other drugs used for insomnia. Using a retrospective, quasi-experimental, difference-in-differences design, the study measured baseline trends, and isolated policy impacts on sedative hypnotic utilization across states and over time for patient subpopulations. The study rationale came from discussions with states participating in the Drug Utilization Review Project (DURP), who expressed concern about the use of sedatives for people with mental illness, and inappropriate use of anti-psychotics for insomnia. Both Colorado and Oregon manage prescription drug coverage using preferred drug lists (PDL). PDLs are like managed formularies in that a committee assesses evidence and advises Medicaid on the medications that should be made available for current practice, based on evidence, FDA indication, and costs. Unlike private health plans with managed formularies, Medicaid cannot have a formulary with discrete limitations on access to medications. Medicaid PDLs list medications to be used first within a drug class (i.e., first-line therapy) with no restrictions on prescription drug coverage. PDLs use policies to influence prescription drug use such as requiring a prescriber to obtain prior authorization, or to prescribe in limited quantities, and patient-targeted PDL policies such as cost-sharing (higher co-payments for non-preferred drugs).

#### Methods

Three distinct Medicaid policies were implemented from 2002 to 2009 on sedative hypnotics: patient cost-sharing, prescriber quantity refill limits, and preferred drug list status/prescriber prior authorization. Retrospective cohorts were created for patients with serious and less severe diagnosed mental illness who met inclusion criteria. Medicaid prescription drug fill claims and eligibility rates by age group and year were acquired for analysis using multivariate methods and time series with segmented regression. Second generation antipsychotics were measured in both states with particular attention to low-dose quetiapine because of its off-label use as a sleep aid.

#### Key Findings

CO and OR baseline rates of psychotropic medication use were substantially different, perhaps because of less-restrictive access policies and attitudes about mental health treatment in OR. Nearly two-thirds of quetiapine use fell below recommended dosing levels, suggesting off-label use for insomnia. Trends in OR suggested reduction in low-dose use after policies were implemented to curb use of antipsychotics for insomnia. Time series analyses of three alternative access restriction policies using segmented regression (unpublished study) found cost-sharing and prior authorization were associated with significant changes in aggregate utilization. Quantity limits alone were not associated with reduced prescription use of newer sedative hypnotics (Campbell et al. 2013; Hartung et al. 2012, 2014).

#### Policy Impact

Colorado used this information in refining its PDL and prior authorization policies, and to identify drug classes in which drug utilization review could be helpful to prescribers and clients. Oregon also used this information to refine its policies around sedative-hypnotics and antipsychotics, and to identify areas in need of further evaluation. The finding of wide variation in prescribing across these two states has led both states to further investigate potential causes of variation (Zerzan et al. 2011).

### GA-SC

Narasimhan and Druss examined the impact of a statewide telepsychiatry program on use of emergency services and costs of services in Georgia and South Carolina. The state initiative targeted for this study was the introduction of telepsychiatry as a new service modality to address the shortage of psychiatric providers and also access to care in rural counties

#### Methods

Individuals treated via telepsychiatry were matched to individuals treated for mental health diagnoses in non-participating hospitals within South Carolina. Regression models were used to assess differences in outpatient follow up, admission following the emergency department (ED) visit, length of stay, inpatient, and total costs.

#### Key Findings

As compared with the control group, the telepsychiatry group was more likely to have successful outpatient mental health follow-up, lower odds of admission and length of stay for the index visit, and lower 30-day inpatient costs than the matched controls.

#### Policy Impact

The findings of improved access enabled the state to make a strong business case for expansion of telepsychiatry. Blue Cross and Blue Shield of South Carolina became champions in leading the way to reimburse telehealth because it would increase access to specialty care for its members. Sustainability efforts are underway.

### NY-PA: Data from Adults

Essock, Donahue, and Stein studied the feasibility and impact of clinical flags developed within Medicaid claims data for New York and Pennsylvania to predict the risk of psychiatric hospitalizations for adults with severe mental illnesses. The purpose of this study was to test the development of claims-based performance measures that could be built into future contracts for managed care to incentivize the engagement of persons in services post-hospitalization, to prevent rapid re-hospitalizations, and to target alternative, less- intensive and less-costly services for state implementation. The study tested how well patterns of service use predicted subsequent high short-term risk of continued psychiatric hospitalizations.

#### Methods

Medicaid claims files were used to identify Medicaid recipients, aged 18–64, with two or more inpatient psychiatric admissions during a target year ending March 31, 2009. Definitions from a quality-improvement initiative were used to identify patterns of inpatient and outpatient service use and prescription fills suggestive of clinical concerns. Generalized estimating equations and Markov models were applied to examine claims through March 2011.

#### Key Findings

Analyses demonstrated that claims data on prior psychiatric hospitalizations can identify Medicaid-enrollees disengaged from treatment (Smith et al. 2014; populations at higher risk of not re-engaging in treatment (Smith et al. 2014), and populations at unusual risk of continued frequent hospitalizations (Stein et al. 2014a, b). Further, as few as 4 months of recent claims data are sufficient for this purpose. Multiple recent hospitalizations, but not failure to use outpatient services nor failure to fill medication prescriptions, were significant predictors of high risk of continued frequent hospitalizations, with odds ratios greater than 4.0 (Stein et al. 2014b).

#### Policy Impact

New York State now routinely uses administrative data to identify individuals at high risk of continued frequent hospitalizations. The NY and PA findings suggested that utilizing recent service use data to identify individuals most in need of services helps to break the cycle of continuing rapid psychiatric readmissions. The NY and PA findings also led to the development and testing of algorithms to avoid determining service eligibility based solely on past service use.

### NY-PA: Data from Children and Youth

Wisdom, Hoagwood, Finnerty, and Stein examined the impact of state-level interventions to improve the quality of psychotropic medication prescribing among publically insured children in NY and in PA. The significant increase in the use of psychotropic medication among children, including increased use of antipsychotics and multiple concurrent medications, raised quality concerns for both states (Essock et al. 2009; Medicaid Medical Directors Learning Network 2010; Kealey et al. 2014). NY engaged mental health clinics in a health information technology (HIT) supported (PSYCKES-Medicaid) continuous quality improvement (CQI) initiative to facilitate reduction of psychotropic polypharmacy among children, and PA introduced a prior authorization policy for the use of antipsychotic medications in children.

#### Methods

The CQI intervention study identified children who received mental health clinic services in New York between 2006 and 2011 and used joinpoint regression analyses to model trends in polypharmacy use among children in CQI participating versus non-participating clinics (Wisdom et al. 2012). The prior authorization study examined the effect of the prior authorization policy on the proportion of Medicaid-enrolled children on antipsychotic medications in PA using a triple-difference strategy including differences between PA and NY, where there was no prior authorization policy; time periods (before and after the introduction of the prior authorization policy in PA), and differences in antidepressant prescribing rates over the same time periods (to control for secular trends in psychotropic prescribing) (Stein et al. 2014a).

#### Key Findings

In NY, a significant shift in polypharmacy trends occurred in the year following the CQI project launch with a significant decrease in use of psychotropic polypharmacy among children in participating clinics, while children in non-participating clinics had a significant increase in use of polypharmacy over the study period. In the prior authorization study, policies were associated with a small but significant reduction in antipsychotic prescribing for children 6–12, but had no impact among 0–5 year olds.

#### Policy Impact

The results of the CQI study suggest that mental health clinics can be successfully engaged in a large scale, HIT-supported quality collaborative and change prescribing practices for children. These findings, along with similar results for adults, supported the continuation and expansion of the HIT CQI program in NY to other quality measures and settings. Further, this approach has been expanded to other states through an Agency for Healthcare Research and Quality funded multi-state quality collaborative (Finnerty et al. 2014). The prior authorization study highlighted that this commonly-used pharmacy benefit management strategy may not work for all populations and medications.

### WA-CT

Morrissey and Frisman examined expedited Medicaid benefits programs in Washington State and Connecticut for released prisoners with severe mental illness. Both states had developed initiatives to improve mental health outcomes and reduce recidivism among released prisoners with severe mental illness. The goal of the study was to assess the impact of expediting benefits on post-prison Medicaid access and uptake, hospitalizations, use of outpatient mental health and substance abuse services, criminal recidivism (re-arrests, jail days, prison incarcerations), and costs.

#### Methods

A quasi-experimental design was used to compare outcomes for prisoners with severe mental illness who received expedited benefits with a control group of prisoners with severe mental illness who did not receive expedited Medicaid. Propensity score weighting was used to balance treatment and control groups on observed baseline demographic and clinical characteristics and behavioral health and criminal justice utilization during a 3-year pre-release period. Linked, administrative data from multiple public sectors were used, and offenders with severe mental illness were followed for up to 36 months after release from prison.

#### Key Findings

In both WA and CT, expedited Medicaid was associated with a greater probability of accessing Medicaid, and quicker Medicaid access after prison release. In addition, expedited Medicaid was associated with a greater probability of accessing mental health services, and quicker access to mental health and medical services. However, there was no effect on criminal justice outcomes as more than 50 % of participants in both groups had at least one re-arrest in the 12 months following index release.

#### Policy Impact

Findings suggest that Medicaid improves mental health service use, but alone, might not be enough to keep prisoners with severe mental illness out of the criminal justice system. Nationally, the implications for the expansion of the Affordable Care Act to justice-involved populations is that simply adding offenders to the Medicaid roles may not be enough to reduce their criminal recidivism. The project also shaped both policy and new research at the state-level, especially in Connecticut, where the state found support for arranging Medicaid benefits for mentally ill prisoners prior to their release, which meant that the program has been sustained. Further, the project demonstrated to stakeholders the value of interagency data mining, supported the expansion of services beyond Medicaid for transitioning prisoners, and facilitated new studies using interagency data.

## Discussion

These projects are examples of the yield that can be expected when state policy leadership and services researchers bring their expertise to bear on policy-relevant questions. Given the profound shifts in state mental health policies as more and more states move to managed care (National Association of State Mental Health Program Directors Research Institute, Inc. 2012), and the restructuring occasioned by healthcare reform, such partnerships will be even more useful for informing state planning and policy making.

These projects were structured as academic-policy partnerships and incorporated ongoing collaborations, frequent communications, and delineation of responsibilities, roles, and partnered decision-making. These partnerships allowed the academic partner to become expert in the complexities, opportunities, and shortcomings of state data files, and the state partner to have the benefit of working with researchers to support development of data-driven policymaking. Having opportunities to feed findings back to stakeholders, to assess hypotheses and interpretations, and to stay abreast of emerging initiatives, fostered engagement between policymakers and researchers and helped ensure that the approaches undertaken would yield findings relevant to that state.

The authors of this paper identified a set of important issues that arose at the system interface of these studies involving state policy and academic research. The issues described below are simultaneously methodologic, administrative, and substantive. We outline these below as a guide for future policy-relevant studies using state administrative data.

### Data Quality

All of the studies used state Medicaid data. As these data are collected for administrative rather than research purposes, researchers had to develop a comfort level in working with information where, often, only approximate answers can be arrived at, even with regard to pressing policy issues. The usability of Medicaid data for policy research varies by state, and researchers need to be aware of the quality of the individual state data they are working with (Byrd and Dodd 2012, 2013). In addition, Medicaid and other claims data are imperfect in that they provide limited information from which to make inferences about the content or quality of services, results of tests, or outcomes of services; in addition, the diagnostic information is generated from routine clinical and billing practices, not research diagnostic interviews. Medicaid claims data reflect service use, not need. In many states, the department of mental health receives information on service use (numerator), but not on the total population of users and non-users (needed for a denominator), hence measures of penetration (e.g., percentage of youth with a mental health visit) can’t be computed.

In comparison to the Medicaid Analytic eXtract (MAX) data maintained by the Center for Medicare and Medicaid Services for policy research purposes, state Medicaid data is more recent by 3 to 4 years, and in some states, is available in nearly real-time. However, state Medicaid data may not be cleaned and standardized across states, meaning researchers need to invest significant time in preparing data for analysis including data cleaning, variable testing, and identifying convergence and divergence among databases across states. For some projects, differences in database quality or content for each two-state study created a great deal of noise, making interpretation more complex. Researchers needed to be flexible, in some cases adapting their study questions or methods as they learned more about the state databases with which they were working (for in-depth discussion of innovative methodological approaches to state policy research, see Duan et al. 2014 and Palinkas et al. 2013).

These experiences speak to the value of including state partners with intimate knowledge not only of state programs but the individual state administrative data files relevant to the study to ensure feasibility and validity of methods. Some projects here incorporated such state data analysts from the outset. The grant projects allowed researchers to become more familiar with individual state databases, and laid the groundwork for future studies and public-academic partnerships.

Because of the variability, state-to-state, in the generosity of Medicaid mental health benefits and regional variation in treatment patterns, generalizability of the findings from any of the state projects to all other states would be problematic. There are also, of course, significant variations in the demographic make-up of the populations across states. Thus, the findings from any of these studies may be applicable to a select number of other states that could be matched on demographic patterns, financing, generosity of benefits, etc. In the selection of state pairings, all investigative teams sought to make meaningful contrasts so that the findings could be applicable to other states. For example, some pairings (e.g., NY and PA) were selected to contrast states with similar coverage. Strategic consideration of the characteristics of the Medicaid benefit package and of state differences needs to be made prior to extrapolation of any of these findings.

### Data Access and Linkage

Multi-system data linkage is easy to envision and difficult to implement. Accessing state data and linking Medicaid data with other state administrative data, or across state Medicaid programs, requires a significant investment of time for both the academic and state partners. Consistent with other reports (Finnerty et al. 2014), in some states, it took university-based investigators half of the 3-year study timetable to develop acceptable data use agreements with state agencies and their contracted data vendors (sometimes separately) to link files with Medicaid claims. In one state, researchers found that key variables, such as eligibility, were not available at the individual level, and that race/ethnicity or cross-system involvement were typically not reported, with the net result that the interagency data was of limited use. States or the federal government could ameliorate this situation by creating the equivalent of advance directives granting academic partners access to such data in an accelerated way when the data will be used for the state’s quality improvement purposes. Further, states need to develop their capacity to track and study populations via administrative data (e.g., from death records, criminal justice, education) in addition to Medicaid so that, when linked to claims data, the composite data will enhance the generalizability and policy relevance of study findings.

Investigators had a much easier time in data access and linkage in states where there were data warehouses, or where there were already-established relationships for multi-agency studies. In Washington State, investigators benefitted from access to a pre-existing data warehouse, maintained by the state, that linked Medicaid claims with a variety of other behavioral health and criminal justice data systems. In Connecticut, these same data were assembled through data use agreements negotiated by the principal investigator, who was the long-term director of research for the state behavioral health agency and an active participant in an interagency data sharing network for multiple prior research studies. In the South Carolina project, investigators were able to use an all-payor dataset as well as Medicaid data.

The take-away message about data access and linkage is that states maintaining data warehouses and those with established university-agency collaborations are better positioned to conduct research using administrative data. States that have invested their own resources in developing university partnerships and data linkages across public sector agencies to support evidence-based management decisions are much better positioned to participate effectively in this type of policy research. Researchers interested in establishing new public-academic partnerships should plan for dedicated time to establish these data sharing agreements and protocols. One benefit of grant-funded public academic partnerships is that once established, data access protocols are easier to expand and sustain, building a foundation for future work (Finnerty et al. 2014).

### Institutional Review Boards (IRB)

The inevitable consequence of attempting to link multi-agency data is that human subject approvals have to be obtained from multiple agency and university IRBs. Ambiguity as to whether the studies were quality improvement or research led to significant (i.e., up to 1 year) IRB approval delays for some groups.

Another factor contributing to research delays had to do with the separation of state staff, who knew Medicaid policies and procedures, from the people at contract organizations, who could read the data file, but knew little about data definitions and codes. This led to discontinuity over time, as some states changed data use agreements, and in some cases, changed contracts about what data would be made available to the researchers. It also led to a loss of institutional memory pertaining to explanations for missing data or spikes in measured observations. Job turnovers and staff changes in some states also led to interruptions in the work.

### Parallel Versus Pooled Analyses

Most of the projects had proposed combining the data from the two comparison states to conduct a pooled analysis, while others proposed parallel analyses from the outset. The pooling of data across states can be cumbersome in the absence of prior experiences with Medicaid and agency coding, defining key parameters (service episodes, eligibility, etc.) consistently in each state, and employing common algorithms to identify comparable samples. During study implementation, however, some state agencies did not approve providing de-identified data to other states for this purpose. As a result, some projects had to construct parallel databases and then conduct separate, parallel analyses. The two approaches—pooling the data or pooling the findings—can lead to equivalent results as long as there is careful alignment and specification of data elements, codes, definitions, and other parameters. An advantage of data pooling is a bump-up in statistical power, which might be important in studies analyzing low-frequency events or small samples. When agency policies prevent combining data across states, or when such pooling would greatly increase the cost and complexity of the task at hand, pooling based upon separate, parallel analyses may be the best and, perhaps only, recourse. In addition, given the large variation observed within and between states, and the challenges in using data across state systems, single state studies should be considered.

### Political Context

State systems are by nature embedded in an operating environment heavily influenced by day-to-day political realities. Change is not only ongoing, but also typically driven by the dominant political culture of the time. Tensions can arise between the pressures of maintaining scientific rigor and meeting the demands of peer review versus the necessity of responding to new policy pressures or initiatives that might divert studies from intended aims. These tensions reflect the different epistemologies and cultures of science versus governing and policy-making. The differing perspectives are an inevitable part of the reality of this kind of academic-policy research partnership.

Even so, conducting rigorous research within a political environment can be facilitated by clear communication and understanding between state and academic partners as to where compromises can and cannot be made on both sides. It requires translation and back-translation between those crafting policy, and those pursuing data-driven answers. Tensions may arise when policymakers need to make quick policy decisions, yet researchers realize that study findings are still emerging and that early findings may not pan out, hence not ready to guide implementation. Policymakers may feel that even preliminary findings offer them more information than they usually have available to inform their decisions. Researchers on the other hand, trained not to draw conclusions early and to look to peer-reviewed publication and confirming studies to increase confidence in conclusions, may feel very uncomfortable sharing preliminary findings or having states act upon them. CQI is a framework that may bridge state and research cultures, and lead to a shared recognition that some questions cannot be answered at all, many are not answered quickly, and that continuous evaluation and review is needed to assess, in real-time, the impact of quality improvement efforts. Frank, open, and honest conversations among the partners about these different pressures help create strong partnerships and high-impact findings focused on state policy priorities.

### Cui Bono*?*

It is appropriate in concluding this brief report to pose the question as to who benefits from this type of research. We believe that there are multiple benefits for different stakeholders from state policy research using administrative data. Benefits accrue to the host states, to researchers committed to developing an evidence-base that can be used to enhance the health of people with severe mental illness or serious emotional/behavioral disorders, and even to the funding agency (in this case, NIMH) for being able to demonstrate its utility in informing state mental health policy (See Table 2). Although the five projects profiled in this report focused primarily on state-level policy issues, state administrative data can also be used at the client, clinician, and program level to inform clinical decision making and quality improvement (Finnerty et al. 2011; PSYCKES-Medicaid 2014) or compare outcomes of treatment interventions at the program level (Morrissey et al. 2013), or at the population level to identify needs and disparities, determine access, and target interventions to address the needs of distinct populations in the mental health and broader health and human services arena.

**Table 2:** Who benefits from use of state administrative data in Policy/Program/Population Health Research?

Federal Research Institutes	States	Researchers
This research…	This research…	This research…
Responds to states and their identified policy issues	Identifies what works for resource allocation decisions	Advances policy/program/population-level comparative effectiveness research
Assesses whether studies using state administrative data can meet peer-review standards	Demonstrates how multiagency administrative data can be organized and analyzed to answer policy/program/population health questions and helps advance state efforts to become learning healthcare organizations	Illustrates how to design, measure and analyze administrative data for policy/program/population health issues
Stimulates cross-state comparisons to advance knowledge about service system design and performance	Fosters academic-state agency collaborations for policy/program/population assessments and gives states access to national experts focused on state priorities	Makes research grant proposals more fundable by using administrative data in lieu of costly prospective data collection
Provides opportunities to support research on delivery system innovations under the Affordable Care Act	Promotes continuous quality improvement to align state and local service providers	Facilitates academic researchers’ understanding of what topics have high policy significance to states, and how to work with state officials to carry out such research
Advances NIMH Strategic Plan	Identifies priority areas for strategic policy development and management via creation of evidence-informed policies	Affords researchers the opportunity to have their work impact public mental health services

Given the publications to date, the current projects demonstrate that grant applications relying on state administrative data can meet the rigors of scientific review. By stimulating cross-state comparisons, federal research institutes or agencies, such as NIMH, can advance knowledge about service system design, performance, and cost-outcomes; they can advance comparative effectiveness assessments at policy, program, and population levels; and they can advance its strategic plan. This type of research can also position federal research agencies to capitalize on the opportunities associated with the Affordable Care Act about efficient service system redesigns.

States, in turn, benefit from supported research done collaboratively with university-based research teams on topics salient to the state mental health authorities and other agency stakeholders. These studies demonstrate how data that states collect for their own administrative and reimbursement purposes can also be used in a rigorous scientific manner to answer questions about what works, for whom, and under what circumstances. Academic partnerships can give states access to national experts focused on questions that are a high priority for states. Importantly, funding for this type of research fosters academic-state agency collaborations that build state capacity to use their data to conduct policy research, help advance state efforts to become learning healthcare organizations, and facilitate growth of a new generation of services research that will be stimulated by the Affordable Care Act.

Lastly, researchers and the field of mental health services and policy research also benefit in important ways. Studies using state administrative data will increasingly become necessary, given declining budgets for services research at the federal level, and reduction of funding for prospective studies of service interventions such as those conducted in the clinical trials tradition. This research provides opportunities for researchers to apply the latest developments in theory and methods with big data sets to answer important questions with immediate relevance to helping people with severe mental illness live more productive lives in their community. In addition, researchers who demonstrate expertise in using state administrative data to answer important questions at the program, population, or policy levels will be much more likely to succeed in the increasingly demanding world of competitive research grant funding.

## Conclusion

The use of state administrative data for generating evidence about public policy issues is both scientifically and ethically valuable. Our combined experiences in developing and maintaining these state-academic partnerships demonstrate that the research-policy-practice gaps that exist can be bridged. We identified five issues that may arise when conducting such studies. The challenges can be significant, yet the yield from this kind of partnered research can have immediate public health benefits. There is also an ethical responsibility to benefit the public by facilitating access to data generated with public dollars, using these data to improve the quality of services for children and adults with mental disorders, and applying the highest-quality scientific methods to ensure accuracy and validity in the interpretations of analyses. States, in collaboration with academic partners, create a fertile laboratory for such work.
